# Coral Reef Disturbance and Recovery Dynamics Differ across Gradients of Localized Stressors in the Mariana Islands

**DOI:** 10.1371/journal.pone.0105731

**Published:** 2014-08-28

**Authors:** Peter Houk, David Benavente, John Iguel, Steven Johnson, Ryan Okano

**Affiliations:** 1 University of Guam Marine Laboratory, UOG Station, Mangilao, Guam; 2 Pacific Marine Resources Institute, Saipan, Northern Mariana Islands; 3 CNMI Bureau of Environmental and Coastal Quality, Saipan, Northern Mariana Islands; California Polytechnic State University, United States of America

## Abstract

The individual contribution of natural disturbances, localized stressors, and environmental regimes upon longer-term reef dynamics remains poorly resolved for many locales despite its significance for management. This study examined coral reefs in the Commonwealth of the Northern Mariana Islands across a 12-year period that included elevated Crown-of-Thorns Starfish densities (COTS) and tropical storms that were drivers of spatially-inconsistent disturbance and recovery patterns. At the island scale, disturbance impacts were highest on Saipan with reduced fish sizes, grazing urchins, and water quality, despite having a more favorable geological foundation for coral growth compared with Rota. However, individual drivers of reef dynamics were better quantified through site-level investigations that built upon island generalizations. While COTS densities were the strongest predictors of coral decline as expected, interactive terms that included wave exposure and size of the overall fish assemblages improved models (R^2^ and AIC values). Both wave exposure and fish size diminished disturbance impacts and had negative associations with COTS. However, contrasting findings emerged when examining net ecological change across the 12-year period. Wave exposure had a ubiquitous, positive influence upon the net change in favorable benthic substrates (i.e. corals and other heavily calcifying substrates, R^2^ = 0.17 for all reeftypes grouped), yet including interactive terms for herbivore size and grazing urchin densities, as well as stratifying by major reeftypes, substantially improved models (R^2^ = 0.21 to 0.89, lower AIC scores). Net changes in coral assemblages (i.e., coral ordination scores) were more sensitive to herbivore size or the water quality proxy acting independently (R^2^ = 0.28 to 0.44). We conclude that COTS densities were the strongest drivers of coral decline, however, net ecological change was most influenced by localized stressors, especially herbivore sizes and grazing urchin densities. Interestingly, fish size, rather than biomass, was consistently a better predictor, supporting allometric, size-and-function relationships of fish assemblages. Management implications are discussed.

## Introduction

A vast array of acute disturbances and chronic stressors threaten coral reefs [1]–[3]. Yet, our knowledge of the role that individual disturbances and stressors play in determining reef dynamics through time remains limited for most locales [4]. One cause of this uncertainty stems from the rarity of ecological datasets that span across sufficient timeframes to capture disturbance and recovery periods, which can help partition the variance associated with population dynamics, and attribute cause, proportionally, to individual stressors. For coral reefs, two putative, localized stressors of primary concern are unsustainable fishing and pollution that may act independently or in combination with disturbance cycles to diminish the growth of reef accreting organisms such as corals [5], [6]. Many studies have compared reefs where high and low human influences existed to generalize how water quality and herbivory have impacted coral growth and resulted in macroalgal replacement [7], [8]. However, less attention has been given to understanding their individual contributions towards growth dynamics outside of laboratory or manipulative settings [9]–[11], and thus, our ability to upscale evidence from manipulative studies to entire reefscapes remains limited. In turn, context-dependent roles of localized stressors continue to be the focus of much research, and varying findings provide support for many ideologies [12]–[14]. When used out of context, or when limited information exists to develop an appropriate context, uncertainty and improperly informed decision making can result.

Acute disturbances such as typhoons, *Acanthaster planci* outbreaks, and climate-induced bleaching are well appreciated for their role in driving coral population dynamics. Disturbance and recovery cycles have traditionally been investigated using coral cover trends, integrated across both local and regional scales, and through time [15], [16]. Yet, coral cover can be an inconsistent metric of recovery due to varying natural environmental regimes, such as wave exposure, that dicate coral growth capacity [17]–[19], and thus the time needed for recovery. Improved assessments of reef condition and calcification potential have emerged from benthic-substrate datasets by simultaneously considering the abundances of macroalgae and other less-calcifying organisms in comparison to corals and other heavily-calcifying organisms [9], [20], [21]. Such integrated metrics can better account for the inherent environmental variation that drives coral cover trajectories, and represents one useful metric of overall recovery potential when comparing across reefs that is furthered within the present study.

In addition to coverage estimates, shifting species abundances [22] and colony-size distributions [23], [24] have also proven to be sensitive indicators of disturbance-and-recovery cycles for coral populations. Studies show that faster growing corals (*Acropora*, *Pocillopora*, *Stylophora*) have lower tolerances to disturbance events, while others (*Porites* and numerous faviids) have a prolonged ability to deal with both acute disturbance and chronic stress [5], [25]–[27]. However, many of the same faster-growing corals may be more resilient (or adaptable) to repeated climate-induced disturbance in the absence of chronic stress [28], [29]. Thus, size distributions and abundance patterns of corals that have low thresholds, fast recovery, and the potential for adaptability provides an additional means of evaluating coral population dynamics [20], [30].

The premise for the present study is that disturbance and recovery are not necessarily co-dependent processes on coral reefs, and by examining them independently with the aid of refined metrics described above, an improved understanding of causation and predicted resilience can emerge [3], [31]. While many studies have described the nature of acute disturbances to reefs from a variety of agents, relatively few have followed recovery dynamics with respect to individual factors. In the Caribbean, Mumby and Harborne [10] reported significantly higher recovery of coral coverage and colony sizes in response to a no-take fishery closure over a 2.5 year period. Similarly, the recovery of coral colony sizes [22], but not recruitment [32], were found to be most heavily dependent upon fishing pressure in Kenya. In American Samoa, Houk et al. [9] found differential recovery of calcifying benthic substrates and coral species evenness to be interactively driven by herbivore biomass and water quality, with their hierarchical influence shifting based upon geological reef settings (i.e., a potential proxy for connectivity with groundwater discharge). In contrast, a meta-analysis of coral reef recovery across all major oceanic basins provided some evidence for unintuitive, reduced recovery rates within fisheries closures following disturbance events [3]. These findings were a perceived artifact of higher pre-disturbance coral coverage within no-take closures, and not attributed to management status. Interestingly, the causes behind recovery trajectories were not consistent across major geographic regions. Clearly recovery is context-dependent with respect to physical settings as well as management regimes [33]–[37]. Considering that predicting reef futures is becoming more of a priority for both local and global management efforts, further study is needed to interpret when and if generalizations may exist, and at what spatial scales they might operate at.

We examined 12-year trends in coral-reef assemblages across fore-reefs in the Commonwealth of the Northern Mariana Islands (CNMI) during a time period when significant disturbance impacts and differential recovery occurred. We first described the nature of *Acanthaster planci* (i.e., Crown-of-Thorn Starfish, COTS) population dynamics during the study period. COTS population trends were augmented with typhoon records to define pre-, during, and post-disturbance time periods across our 12-year study. Coral cover and colony sizes were then evaluated for two study islands that differed in human population, development, and geological setting. Island-scale coral decline and recovery trajectories provided an initial framework for interpreting the nature of coral growth cycles across the study period, and along with previous studies, highlighted primary factors influencing site-specific reef dynamics. Ensuing site-level regressions were performed between coral decline and COTS densities (i.e., the hypothesized, primary disturbance agent), while including key factors that quantified localized stressors and environmental regimes as interactive covariates. Similar site-level regressions were also performed to examine net ecological change across the study period. Combined, our study provided a descriptive framework for disentangling the differences between ‘disturbance impacts’ and ‘net ecological change’, and attributed cause, proportionally, to a suite of factors that help to inform management.

## Methods

### Study location

**Figure 1:**
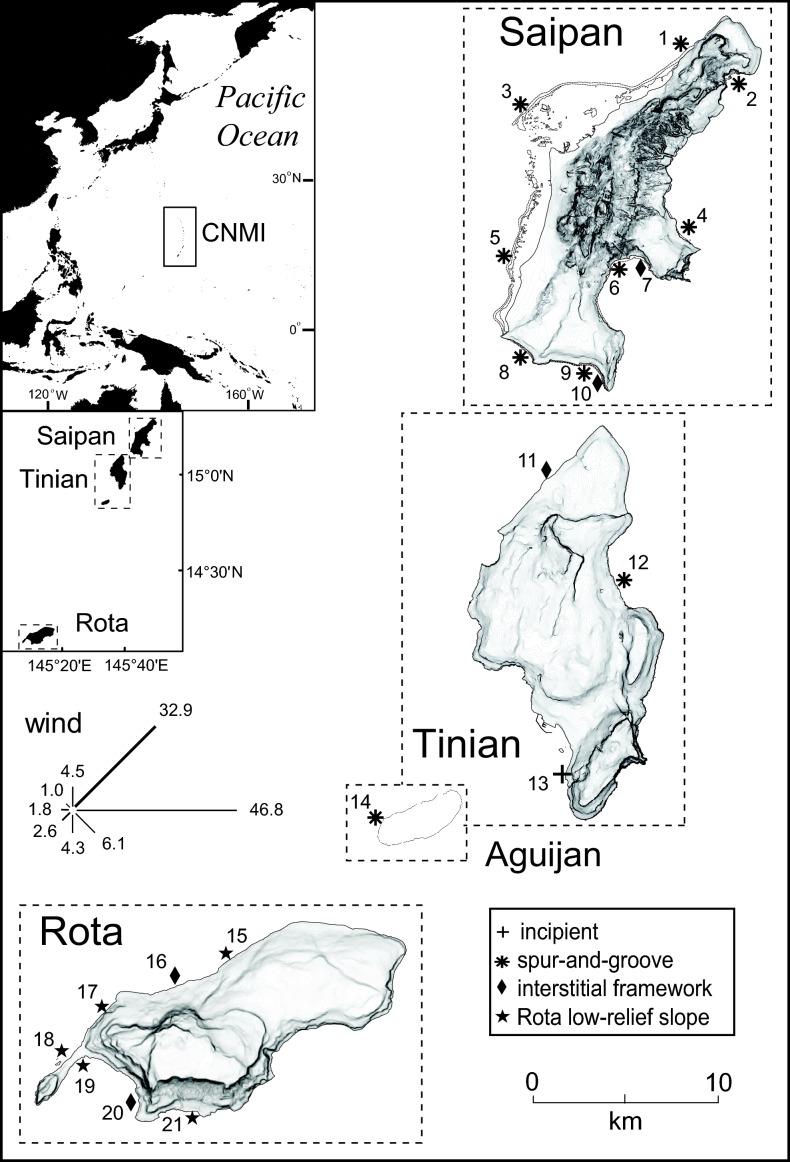
A map of the Western Pacific Ocean and the Commonwealth of the Northern Mariana Islands study islands. Distance between islands is not drawn to scale as dashed boxes indicate individual island entities. Wind vectors show the percent of time that winds originated from each of 8 quadrants (length and corresponding number) as well as the mean annual intensity (thickness). Reeftypes are indicated by symbols referred to in the legend. Topographic lines infer the steepness and size of watersheds.

The Commonwealth of the Northern Mariana Islands (CNMI) represents a series of active volcanic and inactive raised limestone islands located in the Western Pacific Ocean (Figure 1). The present study focused upon the southernmost, inactive limestone islands where the majority of the human population resides. From north to south, the study islands were Saipan (48,220 people, capital island), Tinian (3,136 people), Aguijan (no inhabitants but the nearshore resources are accessed from Saipan and Tinian), and Rota (2,527 people) (Census 2010 statistics, http://commerce.gov.mp/divisions/central-statistics/) (Figure 1).

CNMI’s local monitoring program has been collecting standardized benthic, coral, macroinvertebrate, and fish assemblage data since 2000 [38], [39]. Monitoring designs were stratified based upon geological reeftypes, management status, and watershed development. Geological reeftypes have previously been described with respect to wave exposure and submarine groundwater discharge through karst watersheds that were attributed to specific coral assemblages and reef growth through time [18]. Cumulatively, Houk and van Woesik [18] described four distinct geological reeftypes in the CNMI: 1) optimal spur-and-groove structures, 2) constructional, high-relief interstitial framework, 3) low-relief framework with limited Holocene deposition found only on Rota, and 4) incipient coral assemblages with little to no deposition. Coral coverage, diversity, and evenness peak on the first two, with spur-and-groove structures being the most optimal settings for modern coral assemblage growth.

The most significant acute disturbance since the inception of monitoring efforts has been high *Acanthaster planci* populations (COTS herein) evident between mid-2003 and 2006. In complement to COTS disturbances, several tropical storms passed nearby the study islands during a similar timeframe, with the strongest being Pongsona (passing 40 km south of Rota, 95 knots maximum sustained windspeed) and Chaba (passing over Rota, 118 knot sustained windspeed) in December 2002 and August 2004, respectively. While large wave events were recorded, observations by the authors before and after these storms suggested negligible direct impacts compared with the onset of COTS, examined further within. COTS events became widespread throughout the North Pacific during this timeframe [40]. However, no information exists to understand any potential linkages between the storm events and the subsequent emergence of COTS. Initial examinations were undertaken by coupling COTS abundance and tropical storm histories to define three time periods within the present study *a priori*: pre-, during, and post-disturbance. Subsequently, we quantified how much of the site-level coral decline during the ‘disturbance’ period could be accounted for by COTS.

### Ecological data collection

Data were collected as part of the CNMI Division of Environmental Quality and the CNMI Coastal Resources Management Office coral-reef monitoring program. These programs have the legislative authority to conduct monitoring activities, and given the non-invasive nature of this research, no further permits were required.

Long-term monitoring data have been collected at 1 to 3 year intervals for 25 monitoring stations across the CNMI in most cases, however, longer intervals between repeated site-visits existed in a few instances (Appendix 1). The prerequisite for inclusion in the present study was that coral, benthic, and/or macroinvertebrate data were available for all three timeframes: before, during, and after the disturbance period. Among the 25 monitoring sites, data from 21 met these criteria (Table S1, Figure 1).

Sites were identified by global positioning system coordinates coupled with directional bearings to indicate transect placement. During each survey event, five, 50 m transects were placed at the 8 m depth contour to guide fieldwork. In three instances, homogeneous substrates were not consistently available, and three, 50 m transects were used instead (sites 2, 16, and 18, Figure 1). Benthic substrate abundances were estimated from photographs of 0.5×0.5 m quadrats. In 2000 and 2001, 25 photographs were taken along each transect line, and the substrate under each of 16 data points was identified and recorded. Since 2002, 50 photographs were taken from each transect line, and the substrate under each of 5 data points was recorded. Methods were shifted in order to improve detection limits for temporal change [38]. With respect to the present study, the changes in benthic substrate abundances associated with COTS disturbance were a magnitude of order higher than the expected detection limits based upon 2002 datasets [38]. In all instances, benthic categories chosen for analysis were corals (to genus level), turf algae (non-identifiable turfs typically less than 2 cm), macroalgae (readily identifiable alga typically greater than 2 cm, to genus level if abundant), calcareous encrusting algae known to actively shed epithelial layers or inhibit the survival of juvenile corals (*Peyssonnelia, Pneophyllum*) [41]–[44], crustose coralline algae (CCA) known to promote reef accretion and juvenile coral settlement, sand, and other invertebrates (genus level if abundant). Using these substrate categories, we defined a benthic substrate ratio by the percent cover of heavily calcifying (corals and CCA) versus non-or-less calcifying (turf, shedding-calcareous, macroalgae) substrates.

Coral assemblage data have been collected since 2003 by the same observer using a standard quadrat-based technique. During each survey, 16 replicate 0.5×0.5 m quadrats were haphazardly tossed at equal intervals along the transect lines. All corals whose centerpoint resided within the quadrat boundary were identified, and the maximum diameter, and the diameter perpendicular to the maximum, were recorded. Surface area was calculated from these measurements assuming colonies were elliptical in nature. For three-dimensional corals, measurements were extended along the colony surface area. Coral taxonomy followed Veron [45]. Species-level data were used for analyses of richness and evenness, while species were grouped by ‘sub-genus’ (i.e., genus and growth form, digitate *Acropora*, massive *Porites*) to examine multivariate trends and subsequent species weightings described below.

Macroinvertebrate densities were estimated along individual transect lines (noted above) using 50×4m observation belts. All sea cucumbers, sea urchins, shellfish, and other conspicuous macroinvertebrates were identified to species level and recorded. Count data derived from macroinvertebrate belt transects were used to examine COTS and grazing urchin populations through time.

Fish census protocols were recently added into CNMI long-term monitoring program. The present study used data collected between 2011 and 2012 by a single observer. Fish assemblages were estimated from 12 stationary-point-counts (SPC’s) conducted at equal intervals along the transect lines, following a modified version of [46]. During each SPC, the observer recorded the name (species) and size of all food-fish within a 5 m circular radius for a period of 3-minutes. Food fish were defined by acanthurids, scarids, serranids, carangids, labrids, lethrinids, lutjanids, balistids, kyphosids, mullids, and holocentrids that are a known to be harvested. Sharks were also included. Size data were binned into 5 cm categories (i.e., 12.5–17.5 cm = 15 cm, inferring the size estimates of 13, 14, 15, 16, an 17 were considered as 15 cm), and converted to biomass using coefficients reported in fishbase (www.fishbase.org). Species data were binned into functional categories based upon maximum adult sizes and family level taxonomy for some graphical interpretations and analyses, such as large/small bodied parrotfishes and surgeonfishes, and large/small bodied snappers and groupers. We defined large-and-small-bodied species based upon estimated mean reproductive sizes greater or less than 30 cm, respectively, based upon fishbase records, or the life-history wizard. Planktivores were excluded from the present analyses due to their low abundance in comparison to other trophic groups, and low sample sizes for defining confidence intervals. Last, fish recruits less than 7.5 cm (i.e., 8–12 cm size class bin) were omitted from all analyses to avoid potential bias from differential recruitment with unknown post-settlement mortality dynamics. Recruits comprised less than 3% of the surveyed population.

### Environmental data collection

A proxy for water quality was developed from geographic information system (GIS) layers pertaining to topography, landuse, and human population. Digital elevation models (i.e., topographic data) were used to define watershed boundaries. Landuse data were then overlaid upon the watersheds, and a measure of disturbed land was calculated by combining the coverage of barren land, urbanized vegetation, and developed infrastructure within each watershed (United States Forest Service, http://www.fs.usda.gov/r5). Equal weighting was given to each category because variation in pollution contribution was expected within each. For example, urbanized lands were associated with both septic systems and sewer collection systems that differ with respect to their pollution contribution. Human population density in the watershed adjacent to each site was derived from the 2010 CNMI census. Landuse and human population data were also standardized to weight them equally, and averaged to establish an overall proxy to watershed pollution that represents the simplest assumptions of equal contributions from two known sources of watershed-based pollution. In order to match the low-to-high scale of other localized stressors (i.e., herbivore size), the inverse of the proxy was used in regression modeling.

Wave energy was derived from long-term wind datasets and estimates of fetch [47]. For each site, fetch (i.e., distance of unobstructed open water) was first calculated for 16 radiating lines equally distributed between 20 to 360 degrees. Fully develop sea conditions were considered if unobstructed exposure existed for 20 km or greater. Ten-year windspeed averages were calculated from Saipan airport data (http://www7.ncdc.noaa.gov/), and used as inputs to calculate wave height following Ekebom et al. [47]. Mean height was calculated by:
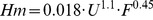
(1)Where Hm is the wave height (m) for each quadrant, U is the windspeed at an elevation of 10 m, and F is the fetch (km). Windspeed corrections for varying elevations were made following Ekebom et al. [47]. Last, wave height was converted to energy following:



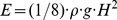
(2)Where ρ is the water density (kg/m^3^), g is the acceleration due to gravity (9.81 m/s^2^), and H is the wave height (m).

## Data Analyses

### Island comparisons

We first generalized disturbance and recovery trajectories for two islands that differed with respect to human population, development, and geological setting, Rota and Saipan. These islands were selected because they both had sufficient sampling effort and spatial coverage over the study period to provide a generalization of temporal reef dynamics (Figure 1, Table S1). Initial, island-scale examinations were conducted for coral cover and colony sizes. Due to varying geological settings, coral growth capacity is inherently lower on Rota compared with Saipan [18], and thus we focused investigations upon the nature and relative rate of decline and recovery, and not absolute values of change. Coral cover data were site-averaged within each of the study time periods, and repeat measures ANOVA tests with post-hoc Tukey’s pairwise tests were conducted between the study periods for each island. Repeat measures ANOVA tests were similarly used to examine coral population density across the disturbance timeframes. Last, differences in coral colony-size distributions were examined using Kolmogorov-Smirnov (K-S) tests that provided a P-value that is generated from the distances between cumulative frequency plots [48].

We next examined sea cucumber and grazing urchin densities across the study periods. Macroinvertebrate count data had zero-inflated, negative binomial distributions that best conformed to zero-inflated, repeat-measures ANOVA models [49]. Zero-inflated models have two distinct parts, one that describes the probability of obtaining a zero count, and one that describes the expected density given a non-zero count. Tests of significance took both simultaneously into account (i.e., hurdle models).

Fish biomass and size were compared between Rota and Saipan within each trophic category noted above: herbivores/detritivores, secondary consumers, and tertiary consumers. Standard comparative tests were used to examine differences in fish size and biomass between the two islands (t-tests, Mann-Whitnet U-test if normality assumptions were not met).

### Site-level analyses

**Figure 2:**
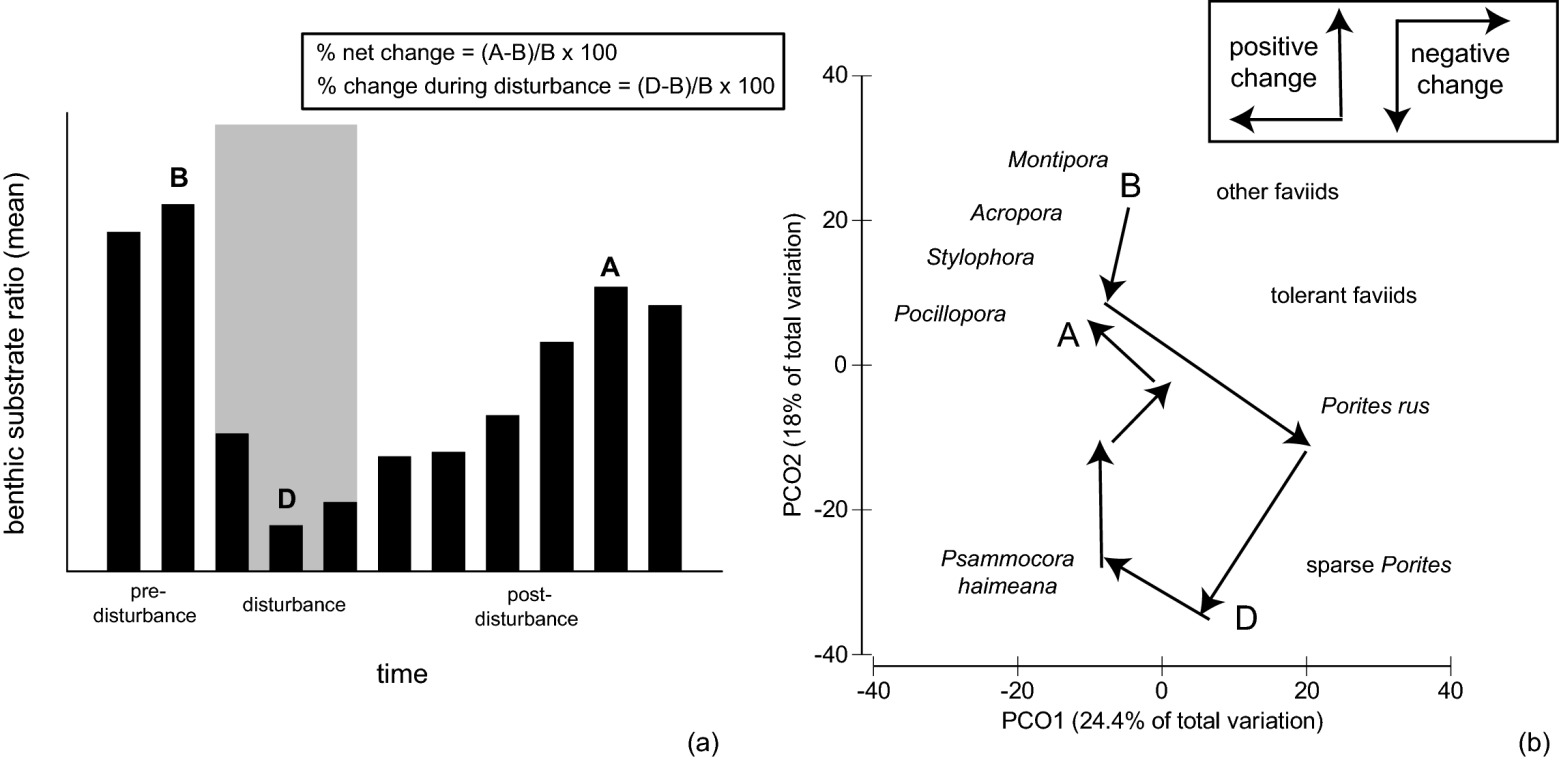
Methods used to calculate the percent decline and net change in (a) the benthic substrate ratio and (b) multivariate measures of the coral assemblages. B, D, A – before, during, and after the disturbance period, respectively. Percent declines were calculated by taking the difference between the minimum and maximum values during and before the disturbance period, and dividing by the pre-disturbance values. Net change values were calculated by taking the difference between maximum values before and after the disturbance period. Values for coral assemblages were calculated based upon their vector magnitude from the origin (0,0), with positive values given for PCO movement towards *Acropora*, *Montipora*, and *Pocillopora* assemblages, and negative values for PCO movement towards tolerant faviids and sparse *Porites*. See methods and Figure 6 for a better description of the corals depicted on the PCO plot.

Deeper investigations of disturbance and recovery trajectories were conducted at the site-level, in order to match the spatial scale that localized stressors and key environmental regimes such as wave exposure operate at. A stepwise regression modeling process was performed to determine the likelihoods and magnitudes of influence for COTS, pertinent localized stressors, and wave exposure. These factors were considered based upon both initial island-scale analyses and previous studies describing their driving influence upon CNMI’s reefs [18], with more details provided below to describe localized stressors. Stepwise modeling to understand the impacts during the disturbance time frame utilized coral cover as the primary, sensitive dependent variable. Modeling to understand net ecological change across the 12-year period used two dependent variables that better described coral reef ‘condition’ and relied less upon coral cover alone, which is known to differ across species and environments [18]. These were: 1) the net change in the benthic substrate ratio, and 2) net change in multivariate coral ordination scores (Figure 2). Benthic and coral ordination metrics were weakly correlated (r<0.4, all comparisons). Coral ordination metrics were calculated based upon two-dimensional principle component ordination plots. Coral species abundance data were log-transformed to produce a Bray-Curtis similarity matrix that quantified multivariate similarities between all sites and years. Similarity matrices were projected in two-dimensional space using principle component ordination (PCO) plots that depicted multivariate differences using axes (i.e., eigenvectors) that hierarchically account for the variation in multi-species datasets (PCO) [50]. Corals that were the strongest drivers of multivariate dissimilarities were overlaid on the plot to indicate the nature of shifting assemblages over the years. The percent net change for coral assemblages represented the two-dimensional PCO-movement (i.e., vector magnitude) away from *Montipora*, *Acropora*, *Stylophora*, and *Pocillopora* assemblages to faviid and *Porites* assemblages, and the magnitude of return proportional to its starting position (Figure 2). These corals cumulatively accounted for the top 30% of the variation in the PCO plot. Percent change values were calculated because they are comparable across sites, whereas absolute values would not be. Second, we used mean values of the benthic substrate ratio defined above. Percent net change was again defined by differences between the post- and pre-disturbance period, divided by the initial pre-disturbance period value (Figure 2). Cumulatively, larger reductions in percent change values indicated a shift to reefs with non-calcifying substrates and smaller, tolerant coral assemblages.

A forward, stepwise regression modeling process was conducted to evaluate the likelihood and magnitude of independent factors in determining the observed biological changes. This process began by searching for factors with a ubiquitous influence upon biological change across all reeftypes. These included COTS in the disturbance timeframe models and wave exposure in the benthic net-change models. No ubiquitous factor was revealed for models describing the net change in coral ordinations, so only individual terms were used. Forward steps were only taken when/if interactive models improved the fit (R^2^-values) and likelihood (AIC-values, described below) of predictions. Interactive combinations were considered because independent variables were all scaled in a consistent, low-to-high manner describing a gradient of weak to strong influences, and are known to have contextual effects that are not independent of one another [9], [33]. If stepwise terms did not improve the model fit and likelihood they were dropped, not presented in the results, and not further considered. This process continued until all interaction terms were examined. We first examined regression models including sites from all reeftypes grouped together, and subsequently examined subsets of the most favorable reef settings for coral growth to determine if herbivory, water quality, or wave exposure might have context-dependent roles that differed in accordance with reeftype. Comparisons of the explanatory power and likelihood of independent variables were only made across models examining the same reeftypes. Our goal was not to suggest that a single “best-fit” model existed, but rather to highlight that several plausible models existed, and provide the details of each. The influence of each factor was assessed based upon: 1) the (added) model fit, 2) overall presence across the suite of models, and 3) the likelihood scores and influence of outliers.

Regression modeling was performed using R [51]. Independent variables were standardized to provide equal scaling, and a constant value was added to make all numbers positive. Positive numbers were required when log-transformations were needed to ensure residual normality. Models were examined for normality using Shapiro-Wilk tests, and ranked based upon their explanatory power (R2-value) and likelihood as measured by Akaike’s Information Criterion (AIC). Lower AIC scores indicated a better fit, based upon the least number of parameters and greatest residual normality that together maximize the probability of given outcomes based upon independent predictors. In all instances, the independent predictors used in interactive regression models were not highly correlated (r<0.30, P>0.05).

Independent variables were selected to represent factors that have a strong influence on CNMI’s coral-reef assemblages and dictate disturbance and recovery dynamics in general. These were COTS densities (i.e., as a disturbance agent), wave exposure, the water quality proxy, mean fish size and biomass, and grazing urchin density (defined by the sum of *Echinothrix*, *Echinometra*, and *Diadema* urchins). When examining models dealing with net ecological change, we used mean herbivore/detritivore fish size and biomass. When examining models dealing with coral cover decline during the disturbance period, we used overall fish size and biomass. This was done in accordance with: 1) the literature describing herbivores as key ecosystem engineers facilitating net recovery dynamics [6], [22], and 2) existing/emerging relationships between enhanced overall fish size/biomass and reduced COTS impact [12], [30], [52].

Last, the present study placed a reliance upon 2010/2011 fish data to predict disturbance and recovery trajectories over the past decade. We do not assume that fish populations were static across the disturbance period, as long-term studies across disturbance events elsewhere clearly define fish assemblage dynamics [53], [54], [55]. However, this approach does assume that site-level relationships were preserved, and relative differences with respect to environmental regimes and/or human exploitation maintained. This was supported by several lines of evidence. Fishery-dependent studies conducted in the CNMI over the past 20 years that showed consistent trends in fish sizes across numerous geographic sectors in the CNMI based upon wave exposure and proximity to human population centers [56], [57], [58], despite evidence for an overall shift (i.e., decline) in several measures of the fishery resource. These studies also highlighted a shift towards increased herbivore dominance and smaller herbivores over the years, suggesting the relevance of herbivore assemblage metrics. In addition, a recent coral reef resilience assessment conducted for 35 sites around Saipan found that wave exposure and MPA-status together were highly correlated with a fishing pressure metric, and fish abundance trends in the present dataset, which were derived from an independent opinion survey taken by CNMI’s resource managers [59]. Beyond CNMI, long-term studies show responses of fish assemblages to disturbances whereby a decline in overall fish diversity, and a decline in the abundance of coral-associated species are most often noted with coral loss [53], [54], [60]. In response to algal growth following disturbances, these studies also show increases in some acanthurids and/or scarids that can respond rapidly to algal substrate availability, with their abundances gradually decreasing to pre-disturbance levels after several, typically 4 to 5, years. Thus, the present regression models were based upon: 1) environmental gradients and management factors known to persist over longer time periods and more extensive spatial gradients than the present study, and 2) fish abundance datasets that were collected 4 to 5 years following the disturbance period when any pulses of herbivores were expected to diminish, and 3) utilized metrics of food-fish size and biomass that were not influenced by smaller, coral-associated species.

## Results

**Figure 3:**
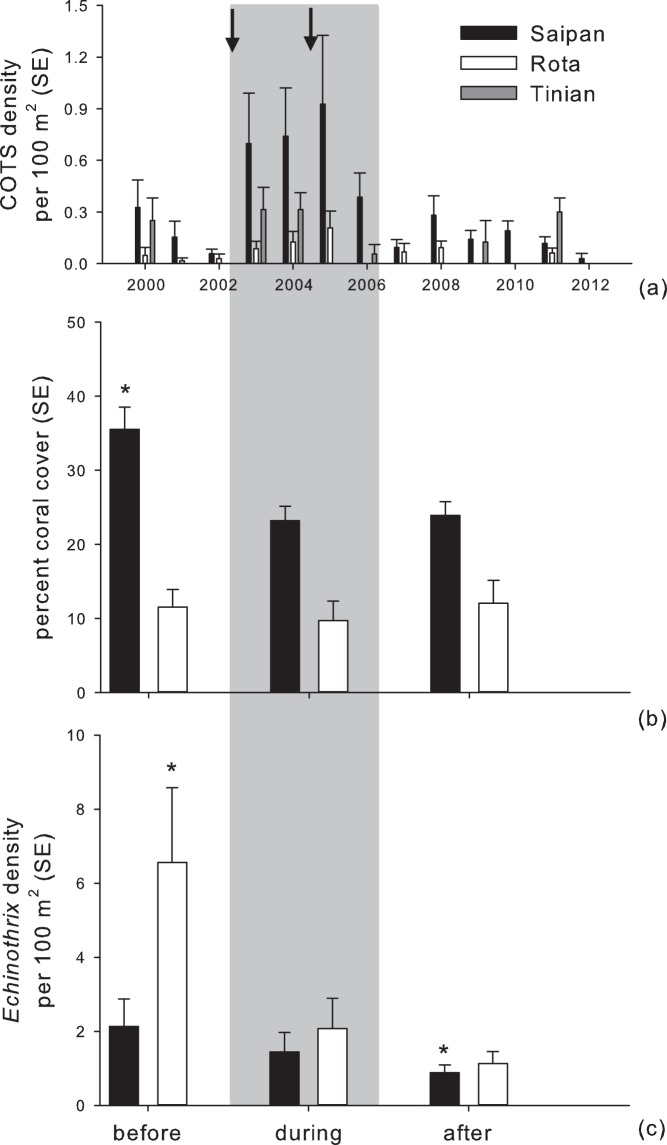
Density of Crown-of-Thorn starfish (COTS) based upon belt-transect data across Saipan, Tinian, and Rota from 2000 to 2012. (a). Densities represent island-based averages that diminish the highest and lowest observations in order to establish patterns across study years. Disturbance reduced coral cover on all islands, but recover trajectories, or the net rate of change, differed by island (b). Coral cover declined on Saipan with no significant recovery (*indicates P<0.05, repeat measures ANOVA and post-hoc tests), while a non-significant decline and recovery was noted on Rota. *Echinothrix* urchins also declined (c) in density during the disturbance period, with a further decline in the years after disturbance (*indicates P<0.05, zero-inflated hurdle models). Black arrows indicate tropical storms that passed by the study islands during the disturbance timeframe (grey rectangle box indicates the disturbance timeframe).

High COTS densities were evident across the CNMI between 2003 and 2006 (Figure 3a, Table S2), concomitant with two tropical storms that passed by the study islands. Together, these findings formed the basis for determining ‘before’, ‘during’, and ‘after’ study periods. Transect-based densities of COTS were largest during the disturbance years on Saipan, however, increased densities were noted across all islands. Following disturbance years, COTS densities declined to pre-disturbance levels for Saipan and Rota, while one unique site on Tinian had an anomalous high density become emergent during 2011 (i.e., site 11 was driving the trend in Figure 3a for Tinian in 2011, Table S2). Cumulatively, COTS densities agreed with 2003 to 2006 as peak disturbance years, but also suggest that Rota and Saipan had similar temporal dynamics, with higher absolute abundances being found on Saipan.

### Island comparisons

**Figure 4:**
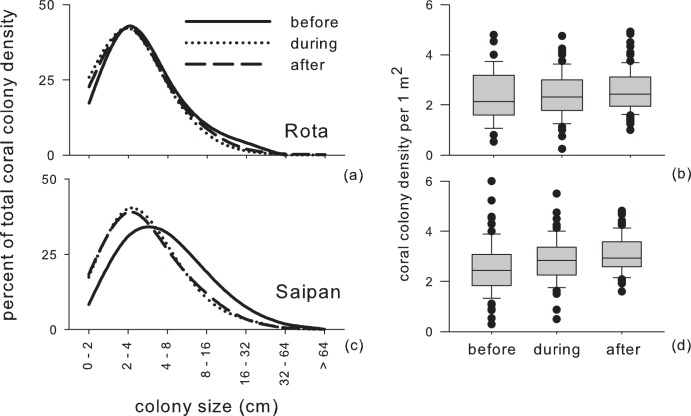
Dynamics of coral colony-size distributions and population densities across the disturbance periods. Rota had a non-significant decline and recovery in colony-size across the study periods (a), as well as a non-significant, sequential increase in population density (b). Reductions in colony-size were evident for Saipan (c) during the COTS period, accompanied by increases in population densities (d), attributed to the emergence of numerous small faviid and *Porites* corals (*indicates P<0.05, repeat measures ANOVA with post-hoc tests, see also Fig. 6).

The disturbance period had a negative impact on coral cover throughout the CNMI, however rates of change were not uniform across the study islands. The largest decline was evident on Saipan, where high human populations and development existed, and smallest on Rota, where human presence and geological foundations for optimal reef growth were lower (Figure 3b). Coral cover declined from 34% to 23% on Saipan (32% decline, F-Statistic = 3.7, P = 0.05, repeat measures ANOVA, pairwise Tukey’s post-hoc q-statistic = 3.6, P = 0.05), with no significant recovery since the disturbance years. Conversely, on Rota where coral cover is naturally lower than Saipan as a consequence of island geology, cover had a non-significant decrease from 11.5% to 9.7%, with recovery back to 12%. Yet, Rota coral cover trends were heavily influenced by a single locality where the coral *Porites rus* is abundant and dominant (Site 20 coral cover 30% whereas all other sites have 10%, Figure 1, Table S2). Re-analysis of the coral cover trends with this site omitted indicated that coral cover had a more substantial decline (8.5% to 4.6%) and recovery back to 7.7% (F-Statistic = 4.0, P = 0.06, repeat measures ANOVA). The impact of disturbances to coral-colony sizes and population densities were also markedly different (Figure 4). Mean coral colony-size on Saipan declined from 7.5 to 5.1cm (P<0.001, K-S test), with no change in the years after disturbance (Figure 3). Demographic changes were complemented by significant increases in coral population density during the disturbance years (F-Statistic = 5.96, P = 0.01, repeat measures ANOVA, pairwise Tukey’s post-hoc q-statistic = 4.8, P<0.05), mainly due to small faviids and *Porites*. In contrast, mean colony size on Rota had a non-significant decline and recovery (4.3 to 3.7cm, followed by a recovery to 4.1cm). Population density comparisons were also non-significant.

Grazing urchin densities declined across the disturbance period on both islands, yet the magnitude of decline was much larger for Rota, where *Echinothrix* densities were reduced from over 6 individuals per 100 m^2^ to less than 2 during the onset of disturbances (Figure 3c, z-statistics = 4.6 and1.8, respectively, for the increased probability of obtaining zero counts and reduction in densities where non-zero counts existed, P<0.01 for both, repeat-measure, zero-inflated hurdle comparisons before and during). Urchin densities were initially lower on Saipan and had a gradual decrease across the study period that was most pronounced in the years following disturbance (z-statistics = 2.8 and1.4, P<0.05, for greater zero counts and reduced densities, respectively, zero-inflated comparisons during and after), representing a timeframe when more abundant COTS existed compared to Rota (Figure 3a). Models indicated that declines were attributed to both higher probabilities of sites with no urchins being present (30% and 38% decline in significant modeled estimates, respectively for Rota and Saipan), and sites with urchins present at lower densities (48% and 58% decline, respectively). No significant differences in sea cucumber densities existed across the study periods.

**Figure 5:**
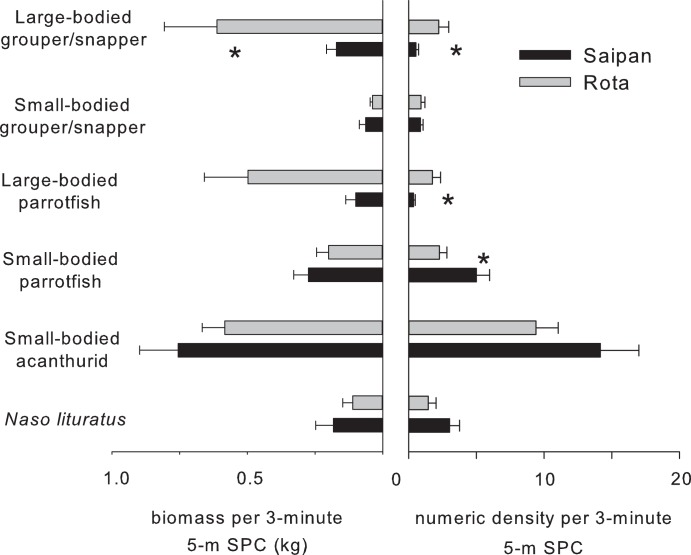
Comparisons of fish biomass and numeric density for several functional fish groups on Saipan and Rota. (*indicates P<0.05, comparative tests described in methods).

Fish biomass and density were consistently greater for large-bodied primary and secondary consumers on Rota compared with Saipan (i.e., species that attain larger reproductive sizes, Figure 5). In contrast, smaller-bodied counterparts were higher in biomass and density on Saipan. These trends were most pronounced for large-bodied groupers, snappers, and parrotfish on Rota, and small-bodied acanthurids and parrotfish on Saipan (P<0.05, comparative t-tests and Mann-Whitney U-tests). The findings support that fewer, large-bodied fish with varying functional roles existed on Saipan, with more numerous, small-bodied species comprising a majority of the biomass.

### Stepwise regression modeling at the site level

**Figure 6:**
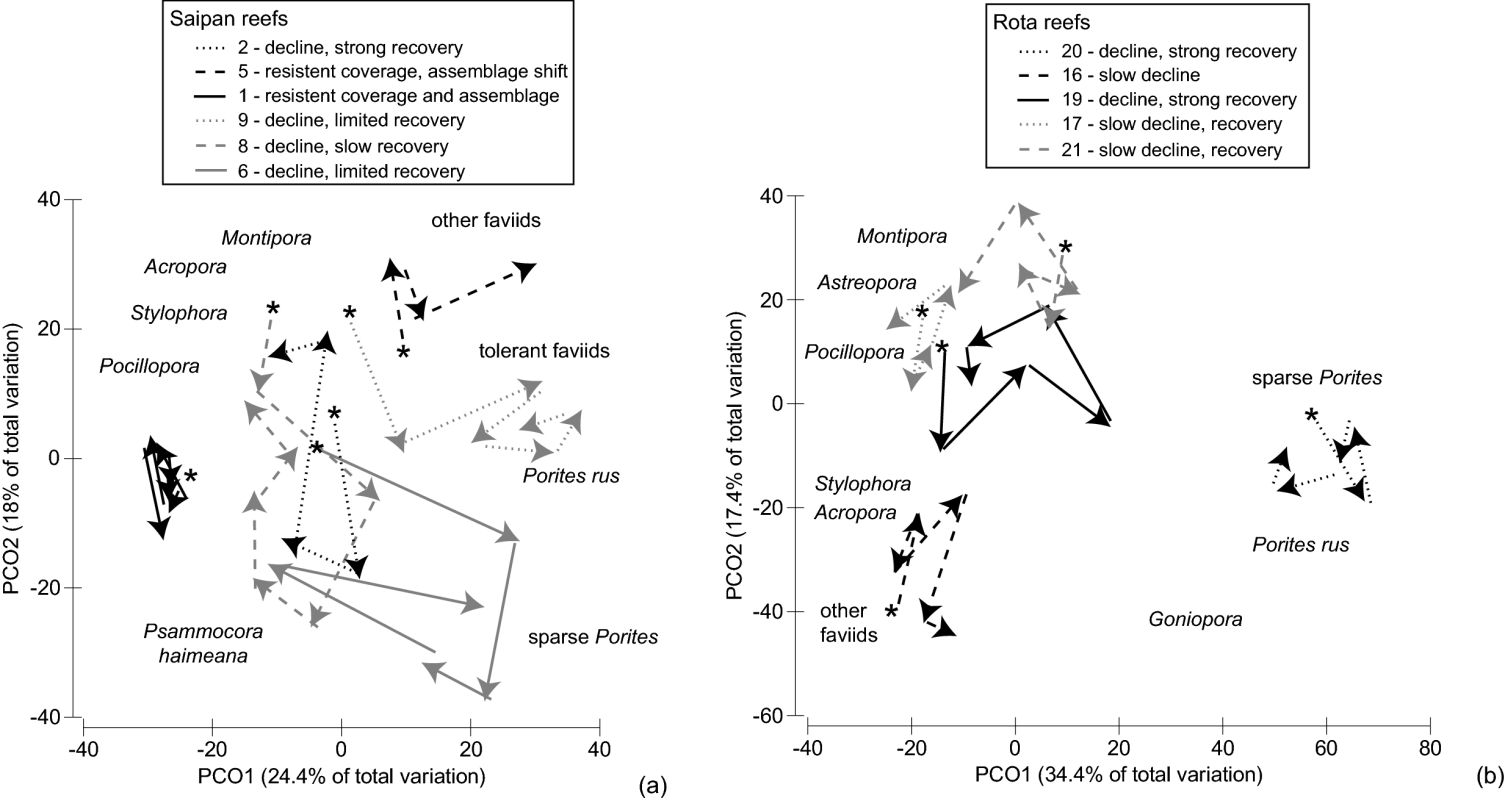
Principle components ordination of coral assemblages for six representative monitoring sites around Saipan (a) and Rota (b). See Figure 1 for site identification and Table S3 for summary statistics. Pre-disturbance assemblages are indicated with an asterisk (*), while vectors depict directional change through time. Sparse *Porites* refers to a dominance of *P. lichen*, *P. vaughani*, and small colonies of other massive species. Tolerant faviids consisted of *Leptastrea purpurea*, *Goniastrea retiformis*, *G. edwardsi*, *Favia matthaii*, *F. pallida*, and *F. favus*. Other faviids consisted of *Favia stelligera*, *Platygyra* spp., *Cyphastrea* spp., and *Favites abdita*.

**Figure 7:**
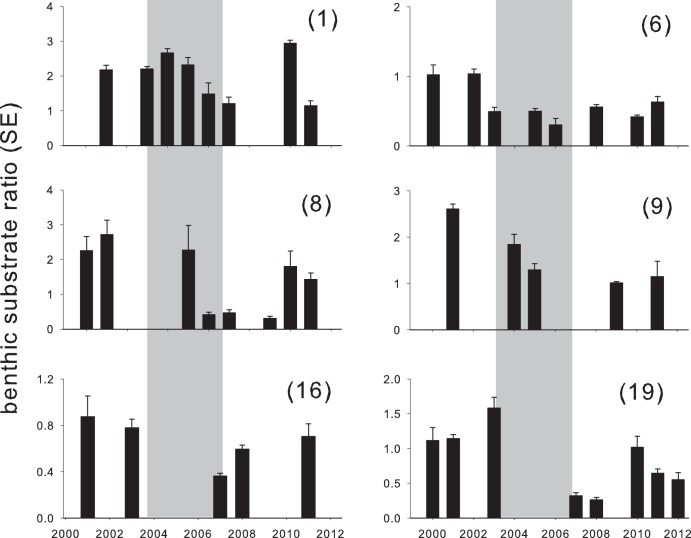
Benthic substrate ratio dynamics for representative monitoring sites around Saipan (sites 1, 9, 8, and 6) and Rota (sites 19 and 16). See Figure 1 for site identification and Table S3 for trends from all sites. Grey bars indicate the disturbance period. Benthic substrate ratios indicate the proportion of heavily-calcifying versus less-or-non-calcifying substrates (*see methods*).

Focusing investigations at the site level revealed that disturbance and recovery cycles varied markedly, with differences attributable, in part, to varying reeftypes and islands that dictated site geomorphology (Figures 6 and 7, Table S3). Percent decline in coral cover during the disturbance period was primarily driven by the observed, transect-based COTS densities regardless of reeftype or island (Table 1). When considering all reeftypes grouped together, only 18% of the variance in coral decline was attributable to COTS (P = 0.06). Yet, the amount of variance explained increased to over 50% when examining the subset of reeftypes with the highest coral growth capacity (52% for interstitial and spur-and-groove reefs combined, and 67% for spur-and-groove reefs alone, P<0.01 for both). The addition of an interaction term describing the mean size of the fish assemblages enhanced the amount of variance accounted for (R^2^ = 0.60 and 0.86, P = 0.009 and 0.005, for interstitial and spur-and-groove reefs combined, and spur-and-groove reefs alone, respectively), while wave exposure improve the fit for spur-and-groove reefs alone (R^2^ = 0.83, P = 0.003, Table 1). Fish size had a negative association with COTS densities for coral-dominated reefs (r = 0.28, Pearson correlation coefficient, interstitial and spur-and-groove reefs combined), while an interactive term of *fish size×wave exposure* had a stronger association with COTS (r = 0.48), highlighting the inter-dependence among these three factors. In sum, COTS were primary predictors of coral decline (i.e., the disturbance period), while COTS densities and coral impacts were diminished with higher wave exposure and larger fish assemblages, especially on reefs with a high capacity for coral growth.

**Table 1:** Stepwise regression models predicting coral decline.

All reeftypes (n = 16)						
*Independent variables*	*Slope*	*SE*	*Intercept*	*R^2^*	*P-Value*	*AIC*
COTS^−1^	6.57	3.20	0.68	0.18	0.06	46.2
**Interstitial and spur-and-groove reefs (n = 12)**						
COTS^−1^	7.84	2.18	0.47	0.52	0.005	25.7
COTS^−1^ × fish_size	1.55	0.43	1.62	0.60	0.009	18.4
**Spur-and-groove reefs (n = 7)**						
COTS^−1^	8.88	2.43	0.23	0.67	0.01	16.8
COTS^−1^ × log(exposure)	8.21	1.50	0.44	0.83	0.003	12.2
COTS^−1^ × fish_size	2.17	0.38	1.30	0.86	0.005	8.6

Summary of forward, stepwise regression models that examined the drivers of coral decline during disturbance years. Methods describe the suite of independent variables examined and the basis for their selection. Significant independent variables presented below include mean *Acanthaster planci* densities during disturbance years (COTS), wave exposure, and mean fish size for all trophic groups combined. COTS densities were inversely scaled for consistency with other localized stressors (i.e., low-bad/high-good). AIC-scores were used to indicate the relative likelihood of models being able to predict outcomes, and are only comparable within each reeftype grouping.

The general response of coral assemblages to disturbance was a decline in *Acropora*, *Montipora*, *Stylophora*, and *Pocillopora* corals, with faviid and *Porites* assemblages becoming emergent, and differential rates of return in the post-disturbance years. Two-dimensional PCO-plots accounted for 40 to 50% of the variance in coral assemblage similarities, and provided a quantitative basis to assess disturbance and net change across individual sites (Figures 2 and 6). Similarly, photo-quadrat data revealed that benthic substrate ratios were sensitive to disturbance (i.e., the ratio of heavily calcifying corals and crustose coralline algae divided by less-calcifying turf, fleshy-coralline, and macroalgae, Figure 7), with differential levels of decline and net change.

In contrast to regression models describing spatial patterns in coral cover declines, COTS densities did not emerge as a significant predictor of net ecological change. This was the case when considering COTS densities during the disturbance timeframe only, or integrated across the entire study period. Stepwise regression modeling for the net change in favorable benthic substrates reported that wave exposure was the strongest individual factor across all reeftypes, accounting for 20% of the variance (P = 0.03, Table 2). The influence of wave exposure grew when considering the subset of reefs with highest coral growth capacity (R^2^ = 0.22, interstitial and spur-and-groove combined, R^2^ = 0.5, spur-and-groove only). Yet, improved model fitting consistently required the inclusion of mean herbivore size and/or grazing urchin density that both increased the explanatory power and likelihood of resultant models. Explanatory power increased by 3%, 16%, and 14% when including herbivore size for all reeftypes, interstitial and spur-and-groove reefs combined, and spur-and-groove reefs alone, respectively (Table 2). Explanatory power increased by 0%, 20%, and 33% when including grazing urchin densities for these same reeftypes, respectively. Last, for coral-dominated reeftypes, including the interaction between herbivore size and grazing urchin density increased model fit by 33%. In all instances, interaction models lowered AIC scores suggesting their greater likelihoods.

**Table 2:** Stepwise regression models predicting net ecological change.

Benthic substrate ratio net change						
All reeftypes (n = 18)						
*Independent variables*	*Slope*	*SE*	*Intercept*	*R^2^*	*P-Value*	*AIC*
log(exposure)	3.81	1.64	−0.76	0.20	0.03	50.8
log(exposure) × herb_size	0.62	0.27	1.33	0.23	0.05	44.6
**Interstitial and spur-and-groove reefs (n = 12)**						
log(exposure)	5.46	2.71	−2.26	0.22	0.07	35.3
log(exposure) × urchin	0.84	0.28	0.74	0.42	0.01	31.7
log(exposure) × herb_size	0.87	0.36	0.85	0.38	0.04	27.4
log(exposure) × herb_size × urchin	0.19	0.06	1.58	0.57	0.01	24.2
**Spur-and-groove reefs (n = 7)**						
log(exposure)	9.23	3.50	−6.06	0.50	0.05	21.8
log(exposure) × herb_size	1.13	0.35	0.40	0.64	0.03	17.6
log(exposure) × urchin	1.11	0.20	0.08	0.83	0.003	14.4
**Coral assemblage net change**						
**All reeftypes (n = 12)**						
herb_size	0.66	0.32	1.37	0.28	0.08	25.0
poll_proxy^−1^	3.89	1.4	7.14	0.38	0.02	32.2
**Spur-and-groove reefs (n = 7)**						
poll_proxy^−1^	4.29	1.71	7.5	0.39	0.04	27.2
herb_size	0.93	0.41	0.64	0.44	0.08	18.9

Summary of forward, stepwise regression models that examined the drivers of net change in the benthic substrate ratio and coral ordination scores across the study period. Methods describe the suite of predictor variables examined and the basis for their selection. Significant independent variables presented below include wave exposure, mean herbivore/detritivore size, mean grazing urchin density, and the water quality proxy. The water quality proxy was inversely scaled (i.e., low-bad/high-good) for consistence with other localized stressors. AIC-scores were used to indicate the relative likelihood of models being able to predict outcomes, and are only comparable within each reeftype grouping.

No single factor consistently emerged as the primary driver of net change in the coral assemblages. Net change was predicted individually and not interactively by both herbivore size and the water quality proxy. When examining sites across all reeftypes, the water quality proxy had a slightly greater explanatory power (R^2^ = 0.28 versus 0.38, herbivore size and water quality proxy, respectively), however the AIC-based likelihood was lower for water quality due to the relatively strong influence of 1 to 2 sites that diminished the normality of residuals (i.e., residuals still met the requirements of normality, but less so as compared with the herbivore size model, reducing the AIC score). The only other notable models emerged when examining spur-and-groove reefs, whereby herbivore size (R^2^ = 0.44, AIC = 18.9) was a slightly better predictor of net coral assemblage change as compared to the water quality proxy (R^2^ = 0.39, AIC = 27.1, Table 1).

## Discussion

Significant coral loss occurred in the CNMI between 2003 and 2006 concurrent with high COTS densities and several typhoons that passed through CNMI. Given that tropical storm paths during these years were consistently in closer proximity to Rota, passing between 60 and 100 km from Saipan, we purport that COTS activity was the primary driver of disturbance that simultaneously impacted the study islands during the mid-2000’s. In support, COTS densities were ubiquitous in models explaining the spatial patterns in coral decline during the disturbance years. However, potential synergies and/or linkages between storm activity and COTS were not approached, and deeper mechanisms may have existed.

While starfish densities were similarly elevated during the disturbance years for two islands that differed in human presence and geology, Rota and Saipan, abundances were highest and most persistent on Saipan. These findings help to explain the greater impacts to coral decline on Saipan. Yet, COTS abundances provided no significant explanation of net recovery patterns to the favorable benthic substrates or coral assemblage dynamics across the entire 12 year study period. We hypothesized the island-scale differences in resistance and recovery were attributable to a suite of factors. First, due to varying island geomorphology, Rota naturally had less coral to begin with (i.e., less prey), including a reduction in preferential prey, *Acropora* and *Montipora* [18]. This situation seems most relevant when interpreting why COTS persistence and coral impacts were diminished on Rota. Second, numerous studies continue to support that fish assemblages comprised of larger individuals across all trophic levels are associated with reduced impacts from COTS disturbances [12], [30], [51], and may help to explain COTS persistence and coral recovery dynamics [10], [22]. Larger biomass and body-size of fish assemblages on Rota compared with Saipan supported this notion. Third, nutrient enrichment from watershed runoff is known to contribute to persistent, localized COTS populations [61], [62]. CNMI water quality reports have consistently found better water quality on Rota over the past decade, with a 50% reduction in bacteria violations on Rota compared with Saipan in 2012 [63]. When considering where persistent populations existed (site 16 on Rota, sites 5, 6, 7, 8, and 10 on Saipan all had densities of 0.2 individuals per 100 m^2^ across several post-disturbance years, Table S2), support for these combined hypotheses grows. Sites 7, 16, and 10 were associated with interstitial framework reefs, posited to have high connections with the karst aquifers compared to others [18] (Figure 1). These sites, in addition to others (5, 6, and 8), all had the smallest overall fish sizes. We synthesize that inherent geological difference as well as localized stressors were influential in describing disturbance dynamics across Rota and Saipan, and utilized site-based analyses to better approach the individual and interactive roles of a suite of factors.

### Site-level drivers of change on CNMI’s reefs

Wave exposure has long been considered to shape modern coral assemblages and reef growth through time in CNMI [64], whereby full exposure to prevailing northeast trade winds has selected against geological reef development through time (i.e., reeftype 4 noted in the methods). Yet, beside the incipient reef development that exists along much of CNMI’s eastern shoreline, significant variation in wave exposure remained among the subset of reeftypes where high coral growth capacity existed, despite having a lower overall magnitude. In fact, this secondary gradient in wave exposure (i.e, low to moderate levels) was the most influential, positive determinant of net benthic substrate change in all reef settings. This may be a result of greater flushing, nutrient transfer rates, and/or the removal of detrital build-up with wave energy [65]. After accounting for wave exposure, the process of grazing, as represented by herbivore/detritivore size and grazing urchin densities, was the strongest and most reliable predictor of favorable benthic substrates based upon their presence across the suite of models, the added variance accounted for, and the improved model likelihood scores (AIC values). These findings were amplified when stratifying by reeftype, and including only reefs with the most favorable geological foundations for coral growth. Water quality emerged as a significant predictor of change for coral assemblages ordinations along with mean herbivore size, however, we purport contextual roles of water quality that are furthered below.

Synthesizing the findings reveals that localized stressors were most influential to reefs with low to moderate wave exposure, and high inherent capacity for coral growth. Given the distribution of geological reef settings in the CNMI (Figure 1), this means that Saipan reefs were weighted disproportionally within the subset of regression models associated with localized stressors, representing 63% of the sites with favorable geological settings (i.e., interstitial or spur-and-groove reefs). Hence, localized stressors were most influential for Saipan, the most populated island where reef-based tourism is centralized, and constitutes a key component of the economy [66].

The present results further an interesting and emerging association between high COTS impacts, smaller-bodied fish assemblages, and low wave exposure. While mechanisms remain unclear and of interest, similar patterns describing diminished COTS impacts with higher fish abundances in successful, no-take marine protected areas have been observed elsewhere [12], [30], [51]. Fish sizes (i.e., herbivores) were also influential to net change metrics, and the collective findings pertaining to the mean sizes of the fish assemblages rather than their biomass resonated well with power laws that describe relationships between body-size, physiology, and function in ecology [67]. Specific to the present study, power laws have been shown to govern numerous physiological traits such as grazing efficiency [68] and reproductive potential [69], whereby a doubling in fish size equates to an exponential increase in function. Thus, even if similar biomass exists, fish assemblages comprised of mainly small-bodied species are expected to have a reduced ecological function within coral-reef food webs [70], [71]. Rasher et al. [72] described that a subset of larger, functionally-dissimilar herbivores play a disproportional role in macroalgal grazing (key constituents include *Chlorurus* spp., *Siganus* spp., and two *Naso* spp., *N. lituratus* and *N. unicornis*), while a suite of other species were more reliant upon generalized detritus and turf grazing from the reef substrate. Species within these functional groups represent highly desirable food fish in CNMI. Market studies have revealed their declining sizes and abundance over the past two decades [55], [56], [57], and also reported a disproportionally small reef-area-per-person, and mean fish size-at-capture, compared to other Micronesia jurisdictions [57]. We reconcile that both size and functional diversity appear to be key attributes of CNMI herbivore assemblages that are sensitive to harvesting pressure, and influential to coral reef recovery patterns, making their improved management desirable for reef futures.

The reduction in *Echinothrix* urchin densities concomitant with COTS disturbances was a novel association to our knowledge. Field observations and photographs provided anecdotal evidence of competition for refuge within the reef matrix, but clearly these relationships remain speculative. In the event that disturbances not only diminished coral cover but structural complexity as well, (i.e., fewer large *Acropora*, *Pocillopora*, *Stylophora* colonies), a decrease in urchin densities was the anticipated ecological response following disturbance [73]. Yet, urchin declines were concomitant with the onset of disturbance, and continued to decline throughout the study period. Given these trends, deeper investigations into the cause(s) of urchin declines seem warranted.

Last, the water quality proxy was an influential driver of net coral assemblage dynamics across the 12 year study period as well, despite its diminished presence across the suite of regression models (i.e., diminished presence in Tables 1 and 2). These findings support previous relationships between water quality proxies and coral species richness in CNMI [18], [20], and reinforce that coral species composition may be a sensitive metric of water quality. It is beyond the purview of this study to formally discuss the linkages between diversity and ecosystem function, however, diversity is well known to facilitate functional redundancy in ecological systems, thereby providing for enhanced resistance and recovery to disturbance. Pollution contribution appeared to be influential to reefs where high human development existed, however, extensive human development was not common within the majority of CNMI’s coastal watersheds. In addition, anchor points in the water quality regression models were driven by the presence of interstitial reefs associated with higher groundwater connectivity (Figure 1, Table 2, and Table S3). We conclude that watershed restoration strategies aimed at improving reef condition might obviously focus upon the largest urban centers, but less obviously, focus upon karst watersheds adjacent to high-value reef assemblages. We note that a major watershed restoration project addressing Laolao Bay (eastern Saipan) remains ongoing, and if successful would serve to address one of the key anchor points.

## Conclusions

Over the past 12 years in CNMI, a period of high COTS densities led to significant coral declines. Yet, the failure of some reefs to recovery was best attributed to localized stressors, which transformed the substrates opened up by coral loss into persistent stands of turf and macroalgae, less conducive for coral replenishment and recovery. Declining trends were strongest for reefs that have favorable geomorphology (i.e., a gently sloping reef foundation), which disproportionally occur on Saipan alongside lower wave exposure. These same reefs represent centers for reef-based tourism that constitutes a major part of CNMI’s economy, highlighting a need to improve upon compromised fish assemblages, grazing urchin populations, and specific localities where water quality concerns exist.

## Supporting Information

Table S1 Monitoring site frequencies. Monitoring frequency for each of the long-term sites incorporated into the present study (*see* *Fig. 1*). Lowercase letters indicate the type of survey conducted in each year: (b) benthic substrate, (i) macroinvertebrate, (c) coral, and (f) fish.
(DOC)

Table S2 Site-based coral coverage and *Acanthaster* density data. Coral coverage and *Acanthaster planci* density summary statistics for each of the long-term monitoring sites incorporated into the present study. Site-based data formed the basis for regression modeling (Table 1). Reeftypes follow: “sg” - optimal spur-and-groove structures, “int” - high-relief, interstitial framework, “rot” - low relief Holocene framework found on Rota only, and “pl” - incipient coral assemblages residing upon a Pleistocene basement (*see methods*).
(DOC)

Table S3 Site-based summary statistics for regression models. Summary statistics for each of the long-term monitoring sites incorporated into the present study. Site-based data formed the basis for regression modeling. Dependent variables included the net change in the benthic substrate ratio and coral assemblages, noted as the sum of the percent decline (−) and subsequent recovery (+) of these ecological metrics (*see methods*). Reeftypes follow: “sg” - optimal spur-and-groove structures, “int” - high-relief, interstitial framework, “rot” - low relief Holocene framework found on Rota only, and “pl” - incipient coral assemblages residing upon a Pleistocene basement (*see methods*).
(DOC)
