# Correction: Coral Reef Disturbance and Recovery Dynamics Differ across Gradients of Localized Stressors in the Mariana Islands

**DOI:** 10.1371/journal.pone.0110068

**Published:** 2014-09-30

**Authors:** The PLOS ONE Staff

## Notice of Republication

This article was republished on September 12, 2014, to correct typesetting errors with the figures. The publisher apologizes for these errors. Please download this article again to view the corrected figures. The originally published, uncorrected article and the republished, corrected article are provided here for reference.

## Supporting Information

File S1 **Originally published, uncorrected article**
PDF

File S2 **Republished, corrected article**
PDF
