# Supplementary material for: Coral Reef Disturbance and Recovery Dynamics Differ across Gradients of Localized Stressors in the Mariana Islands
Source: PLoS One. 2014 Aug 28;9(8):e105731. doi: 10.1371/journal.pone.0105731 (PMC4148314; doi:10.1371/journal.pone.0105731)
Supplement: Table S1 — Monitoring site frequencies. Monitoring frequency for each of the long-term sites incorporated into the present study (see Fig. 1 ). Lowercase letters indicate the type of survey conducted in each year: (b) benthic substrate, (i) macroinvertebrate, (c) coral, and (f) fish. (DOC) [file pone.0105731.s001.doc]

**Table S1 title: Monitoring site frequencies.**

Table S1. Monitoring frequency for each of the long-term sites incorporated into the present study (*see Figure 1*). Lowercase letters indicate the type of survey conducted in each year: (b) benthic substrate, (i) macroinvertebrate, (c) coral, and (f) fish.

| *Site* | *2000* | *2001* | *2002* | *2003* | *2004* | *2005* | *2006* | *2007* | *2008* | *2009* | *2010* | *2011* | *2012* | |
| --- | --- | --- | --- | --- | --- | --- | --- | --- | --- | --- | --- | --- | --- | --- |
| **1** |  | b, i | i | b, c, i | b, c, i | b, c, i | b, c, i | b, c, i |  |  | b, c, i | b, c, i, f |  |  |
| **2** | b, i |  | i | c |  | c |  | b, c, i |  |  | b, c, i |  | b, c, i, f |  |
| **3** |  | b, i | i | b, c, i |  |  | b, c, i |  |  | b, c, i |  | b, c, i, f |  |  |
| **4** |  |  |  |  | b, i | c |  |  |  |  | b, c, i |  | c, i, f |  |
| **5** | b, i | b, i |  | c, i |  | b, c, i |  |  | b, c, i | b, c, i |  | b, c, i, f | i |  |
| **6** | b |  | b, i | b, c, i |  | b, c, i | b, c, i |  | b, c, i |  | b, c, i |  | b, c, f |  |
| **7** | b, i | i | b, i | c, i | b, c, i | i | b, c, i |  | b, c, i | b, c, i |  | b, c, i, f |  |  |
| **8** | b, i | b, i |  | c, i |  | b, c, i | b, c, i | b, c, i |  | b, c, i | b, c, i | b, c, i, f | c |  |
| **9** | i | b, i |  | c, i | b, c, i | b, c, i |  |  |  | b, c, i | c | b, c, i | c, i, f |  |
| **10** | i | b, i |  | c, i | b, c, i | b, c, i |  | b, c, i | b, c, i |  | b, c, i | b, c, i | c, f |  |
| **11** |  | b |  | i | b, c, i | b, c, i | c, i |  |  | b, c, i |  | b, c, i | i, f |  |
| **12** |  |  |  | b, c |  | b, c |  |  | b, c, i |  |  | b, c, i, f | i |  |
| **13** | b, i |  | b, i | c | i | b, c |  |  | b, c, i |  | b, c |  | b, c, i, f |  |
| **14** |  | b, i | b, i | c |  | c |  |  |  |  |  |  | c, f |  |
| **15** |  | b, i | i | c, i | c, i |  |  | b, c, i |  |  |  | b, c, i | b, c, i, f |  |
| **16** | i | b, i |  | b, c, i | c, i | c, i |  | b, c, i | b, c, i |  |  | b, c, i |  |  |
| **17** |  |  |  | c |  | b, c, i |  |  | b, c, i |  |  | b, c, i | b, c, i, f |  |
| **18** | b, i |  | b, i | b, c, i | c, i |  |  | b, c, i |  |  |  | b, c, i | b, c, i, f |  |
| **19** | b, i | b, i | i | b, c, i | c, i | c, i |  | b, c, i | b, c, i |  | b | b, c, i | b, c, i, f |  |
| **20** | b, i | b, i | i | b, c, i | c, i | c, i |  | b, c, i | b, c, i |  | b | b, c, i | b, c, i, f |  |
| **21** | b, i | b, i |  | i | b, c, i | b, c, i |  | b, c, i | b, c, i |  | b | b, c, i | b, c, i, f |  |
