# Supplementary material for: Coral Reef Disturbance and Recovery Dynamics Differ across Gradients of Localized Stressors in the Mariana Islands
Source: PLoS One. 2014 Aug 28;9(8):e105731. doi: 10.1371/journal.pone.0105731 (PMC4148314; doi:10.1371/journal.pone.0105731)
Supplement: Table S2 — Site-based coral coverage and Acanthaster density data. Coral coverage and Acanthaster planci density summary statistics for each of the long-term monitoring sites incorporated into the present study. Site-based data formed the basis for regression modeling (Table 1). Reeftypes follow: “sg” - optimal spur-and-groove structures, “int” - high-relief, interstitial framework, “rot” - low relief Holocene framework found on Rota only, and “pl” - incipient coral assemblages residing upon a Pleistocene basement (see methods). (DOC) [file pone.0105731.s002.doc]

**Table S2 title: Site-based coral coverage and *Acanthaster* density data.**

Table S2. Coral coverage and *Acanthaster planci* density summary statistics for each of the long-term monitoring sites incorporated into the present study. Site-based data formed the basis for regression modeling (Table 1). Reeftypes follow: “sg” - optimal spur-and-groove structures, “int” - high-relief, interstitial framework, “rot” - low relief Holocene framework found on Rota only, and “pl” - incipient coral assemblages residing upon a Pleistocene basement (*see methods*).

| *Site* | *Island* | *Reeftype* | *Coral Coverage* | | | *Acanthaster planci (density per 100m^2^) (SD)* | | |
| --- | --- | --- | --- | --- | --- | --- | --- | --- |
|  |  |  | *Before* | *During* | *After* | *Before* | *During* | *After* |
| **1** | Saipan | sg | 29.5 (4.7) | 27.4 (4.6) | 32.1 (6.1) | 0.00 | 0.00 | 0.07 (0.28) |
| **2** | Saipan | sg | 18.9 (2.4) | 18.7 (3.0) | 32.9 (5.4) | 0.00 | 0.33 (0.52) | 0.11 (0.19) |
| **3** | Saipan | sg | 31.1 (5.5) | 33.3 (4.1) | 30.9 (3.7) | 0.00 | 0.00 | 0.00 |
| **4** | Saipan | sg | -- | 19.3 (4.3) | 34.5 (3.4) | -- | 0.00 | 0.00 |
| **5** | Saipan | sg | 48.4 (8.8) | 32.3 (2.7) | 35.9 (10.4) | 0.08 (0.28) | 0.11 (0.33) | 0.38 (0.54) |
| **6** | Saipan | sg | 36.4 (6.2) | 11.5 (2.9) | 11.3 (2.6) | 0.0 | 4.28 (6.8) | 0.78 (0.83) |
| **7** | Saipan | int | 27.4 (5.9) | 25.1 (6.4) | 24.3 (6.9) | 0.50 (0.80) | 1.14 (1.28) | 0.54 (0.66) |
| **8** | Saipan | sg | 47.4 (6.2) | 20.3 (11.2) | 11.3 (3.4) | 0.0 | 9.5 (8.4) | 0.05 (0.22) |
| **9** | Saipan | sg | 42.1 (6.0) | 23.1 (8.4) | 21.3 (7.0) | 0.0 | 3.22 (3.96) | 0.38 (0.77) |
| **10** | Saipan | int | 20.7 (3.9) | 18.0 (2.4) | 22.3 (7.7) | 2.01 (2.34) | 0.11 (0.33) | 0.61 (0.77) |
| **11** | Tinian | int | 8.1 (2.1) | 4.7 (0.9) | 11.2 (2.5) | -- | 0.40 (0.68) | 0.56 (0.53) |
| **12** | Tinian | sg | -- | 22.4 (3.6) | 26.3 (3.1) | -- | -- | 0.22 (0.44) |
| **13** | Tinian | pl | 12.3 (3.7) | 5.1 (1.9) | 10.6 (3.1) | 0.0 | 0.8 (1.09) | 0.0 |
| **14** | Aguijan | sg | 40.6 (5.7) | -- | 35.2 (7.0) | 0.0 | -- | -- |
| **15** | Rota | rot | 8.6 (2.4) | -- | 12.4 (3.5) | 0.0 | 0.0 | 0.0 |
| **16** | Rota | int | 9.0 (1.6) | 5.9 (1.2) | 10.5 (3.3) | 0.0 | 0.33 (0.58) | 0.0 |
| **17** | Rota | rot | -- | 5.2 (0.4) | 6.0 (1.9) | -- | 2.25 (2.06) | 0.11 (0.33) |
| **18** | Rota | rot | 7.7 (1.8) | 7.8 (1.8) | 10.0 (2.5) | 0.0 | 0.5 (0.84) | 0.0 |
| **19** | Rota | rot | 8.4 (1.9) | 5.0 (1.7) | 5.5 (2.2) | 0.15 (0.37) | 0.2 (0.45) | 0.17 (0.51) |
| **20** | Rota | int | 26.4 (5.3) | 22.2 (10.0) | 34.2 (13.3) | 0.0 | 0.27 (0.46) | 0.05 (0.23) |
| **21** | Rota | rot | 8.2 (1.8) | 1.3 (0.8) | 6.4 (1.6) | 0.0 | 0.33 (0.72) | 0.00 |
