# Supplementary material for: Coral Reef Disturbance and Recovery Dynamics Differ across Gradients of Localized Stressors in the Mariana Islands
Source: PLoS One. 2014 Aug 28;9(8):e105731. doi: 10.1371/journal.pone.0105731 (PMC4148314; doi:10.1371/journal.pone.0105731)
Supplement: Table S3 — Site-based summary statistics for regression models. Summary statistics for each of the long-term monitoring sites incorporated into the present study. Site-based data formed the basis for regression modeling. Dependent variables included the net change in the benthic substrate ratio and coral assemblages, noted as the sum of the percent decline (−) and subsequent recovery (+) of these ecological metrics (see methods). Reeftypes follow: “sg” - optimal spur-and-groove structures, “int” - high-relief, interstitial framework, “rot” - low relief Holocene framework found on Rota only, and “pl” - incipient coral assemblages residing upon a Pleistocene basement (see methods). (DOC) [file pone.0105731.s003.doc]

**Table S3 title: Site-based summary statistics for regression models.**

Table S3. Summary statistics for each of the long-term monitoring sites incorporated into the present study. Site-based data formed the basis for regression modeling. Dependent variables included the net change in the benthic substrate ratio and coral assemblages, noted as the sum of the percent decline (-) and subsequent recovery (+) of these ecological metrics (*see methods*). Reeftypes follow: “sg” - optimal spur-and-groove structures, “int” - high-relief, interstitial framework, “rot” - low relief Holocene framework found on Rota only, and “pl” - incipient coral assemblages residing upon a Pleistocene basement (*see methods*).

| *Site* | *Reeftype* | *Watershed size (km^2^)* | *Disturbed land (km^2^)* | *Human population* | *Wave energy (J/m^3^)* | *Herbivore size (cm)* | *Herbivore biomass (kg per SPC)* | *Sea cucumber density (per 100 m^2^)* | *Grazing urchin density (per 100m^2^)* | *Benthic substrate ratio* | | *Coral assemblage PCO scores* | |
| --- | --- | --- | --- | --- | --- | --- | --- | --- | --- | --- | --- | --- | --- |
|  |  |  |  |  |  |  |  |  |  | *% decline* | *% net change* | *% decline* | *% net change* |
| **1** | sg | 0.05 | 0.02 | 0 | 202 | 11.6 | 2.33 | 0.53 | 4.45 | -55.1 | 9.7 | -0.01 | 4.9 |
| **2** | sg | 1.06 | 0.09 | 20 | 330 | 13.54 | 1.89 | 29.24 | 7.26 | -58.4 | 54.7 | -40.3 | 28.1 |
| **3** | sg | 0 | 0 | 0 | 451 | -- | -- | 7.13 | 2.39 | 30.0 | 13.8 | -22.7 | 16.3 |
| **4** | sg | 7.8 | 3.85 | 450 | 1730 | 14.95 | 0.78 | 6.3 | 4.17 | -- | -- | -- | -- |
| **5** | sg | 0 | 0 | 0 | 113 | 10.28 | 0.36 | 1.11 | 2.94 | -68.3 | -34.3 | -- | -- |
| **6** | sg | 1.56 | 0.29 | 330 | 210 | 10.2 | 0.66 | 2.32 | 2.81 | -71.1 | -39.4 | -86.6 | -65.8 |
| **7** | int | 1.2 | 0.3 | 270 | 82 | -- | -- | 4.88 | 5.15 | -53.5 | -23.5 | -144.2 | -111.3 |
| **8** | sg | 0.35 | 0.19 | 105 | 143 | 10.57 | 0.43 | 9.81 | 1.11 | -84.9 | -33.9 | -66.7 | -11.1 |
| **9** | sg | 1.24 | 0.26 | 25 | 103 | 11.82 | 1.92 | 1.03 | 1.19 | -50.6 | -56.3 | -67.8 | -69.8 |
| **10** | int | 1.2 | 0.01 | 0 | 57 | 11.25 | 1.09 | 3.7 | 2.98 | -53.1 | -5.8 | -- | -- |
| **11** | int | 1.3 | 0.1 | 0 | 130 | 12.54 | 1.19 | 12.76 | 3.88 | -51.6 | -50.9 | -- | -- |
| **12** | sg | 1.3 | 0.1 | 10 | 1422 | 14.08 | 0.65 | 0.78 | 4.39 | -- | -- | -- | -- |
| **13** | pl | 0.65 | 0.1 | 30 | 143 | 11.28 | 0.5 | 2.52 | 4.14 | -50.6 | -66.1 | -- | -- |
| **14** | sg | 0.3 | 0.04 | 0 | 205 | 15.12 | 1.8 | 0.1 | 0.75 | -- | -- | -- | -- |
| **15** | rot | 1 | 0.35 | 50 | 455 | 12.43 | 0.79 | 0.22 | 4.17 | -- | 19.2 | -- | -- |
| **16** | int | 2.9 | 0.7 | 30 | 321 | -- | -- | 2.44 | 3.9 | -58.6 | -19.5 | -19.6 | -20.3 |
| **17** | rot | 0.5 | 0.15 | 25 | 174 | 11.86 | 0.86 | 0.23 | 1.62 | -70.8 | -13.8 | -5.1 | -1.4 |
| **18** | rot | 0.08 | 0.05 | 30 | 172 | 11.32 | 0.91 | 1.23 | 8.75 | -59.1 | 15.9 | -31.1 | 20.0 |
| **19** | rot | 0.13 | 0.07 | 200 | 138 | 13.78 | 2.3 | 4.23 | 10.3 | -80.3 | -36.1 | -41.7 | 0.8 |
| **20** | int | 0.4 | 0.1 | 35 | 119 | 13.46 | 1.75 | 0.87 | 1.92 | -13.8 | 5.1 | -96.5 | 99.8 |
| **21** | rot | 1.7 | 0.6 | 20 | 725 | 12.66 | 1.4 | 2.62 | 3.9 | -52.9 | 2.9 | -- | -- |
